# An optimized protocol for the generation and monitoring of conditional orthotopic lung cancer in the KP mouse model using an adeno-associated virus vector compatible with biosafety level 1

**DOI:** 10.1007/s00262-023-03542-z

**Published:** 2023-10-05

**Authors:** Haibin Deng, Huixiang Ge, Christelle Dubey, Tereza Losmanova, Michaela Medová, Georgia Konstantinidou, Seyran Mathilde Mutlu, Fabienne Esther Birrer, Tess Melinda Brodie, Deborah Stroka, Wenxiang Wang, Ren-Wang Peng, Patrick Dorn, Thomas Michael Marti

**Affiliations:** 1 Department of General Thoracic Surgery, Inselspital, Bern University Hospital, University of Bern, Murtenstrasse 28, 3008 Bern, Switzerland; 2 Department for BioMedical Research, University of Bern, Bern, Switzerland; 3 Graduate School of Cellular and Biomedical Sciences, University of Bern, Bern, Switzerland; 4 Institute of Pathology, University of Bern, Bern, Switzerland; 5 Department of Radiation Oncology, Inselspital, Bern University Hospital, University of Bern, Bern, Switzerland; 6 Institute of Pharmacology, University of Bern, Bern, Switzerland; 7 Department of Pulmonary Medicine, Inselspital, Bern University Hospital, University of Bern, Bern, Switzerland; 8 Department of Visceral Surgery and Medicine, Inselspital, Bern University Hospital, University of Bern, Bern, Switzerland; 9 Thoracic Surgery Department 2, Hunan Cancer Hospital and the Affiliated Cancer Hospital of Xiangya School of Medicine, Central South University, Changsha, 410013 Hunan China; 10 Hunan Key Laboratory of Translational Radiation Oncology, Hunan Cancer Hospital and the Affiliated Cancer Hospital of Xiangya School of Medicine, Central South University, Changsha, 410013 China

**Keywords:** Lung adenocarcinoma, Mouse model, BSL-1, Intratracheal instillation, AAV vector, Cre-expressing vector

## Abstract

**Background:**

The inducible Kras/p53 lung adenocarcinoma mouse model, which faithfully recapitulates human disease, is routinely initiated by the intratracheal instillation of a virus-based Cre recombinase delivery system. Handling virus-based delivery systems requires elevated biosafety levels, e.g., biosafety level 2 (BSL-2). However, in experimental animal research facilities, following exposure to viral vectors in a BSL-2 environment, rodents may not be reclassified to BSL-1 according to standard practice, preventing access to small animal micro-computed tomography (micro-CT) scanners that are typically housed in general access areas such as BSL-1 rooms. Therefore, our goal was to adapt the protocol so that the Cre-induced KP mouse model could be handled under BSL-1 conditions during the entire procedure.

**Results:**

The Kras-Lox-STOP-Lox-G12D/p53 flox/flox (KP)-based lung adenocarcinoma mouse model was activated by intratracheal instillation of either an adenoviral-based or a gutless, adeno-associated viral-based Cre delivery system. Tumor growth was monitored over time by micro-CT. We have successfully substituted the virus-based Cre delivery system with a commercially available, gutless, adeno-associated, Cre-expressing vector that allows the KP mouse model to be handled and imaged in a BSL-1 facility. By optimizing the anesthesia protocol and switching to a microscope-guided vector instillation procedure, productivity was increased and procedure-related complications were significantly reduced. In addition, repeated micro-CT analysis of individual animals allowed us to monitor tumor growth longitudinally, dramatically reducing the number of animals required per experiment. Finally, we documented the evolution of tumor volume for different doses, which revealed that individual tumor nodules induced by low-titer AAV-Cre transductions can be monitored over time by micro-CT.

**Conclusion:**

Modifications to the anesthesia and instillation protocols increased the productivity of the original KP protocol. In addition, the switch to a gutless, adeno-associated, Cre-expressing vector allowed longitudinal monitoring of tumor growth under BSL-1 conditions, significantly reducing the number of animals required for an experiment, in line with the 3R principles.

**Supplementary Information:**

The online version contains supplementary material available at 10.1007/s00262-023-03542-z.

## Background

### The broad applicability of the KP model

Orthotopic, genetically engineered mouse models of cancer provide the basis to study tumor initiation, progression, and treatment options in the pathophysiologically relevant tumor microenvironment [27]. Lung cancer is the leading cause of cancer-related deaths worldwide, with more than 80% of lung tumors being non-small cell lung cancer (NSCLC). Oncogenic *KRAS* mutations are frequent in lung and other solid tumors, with a prevalence of approximately 30% in NSCLC adenocarcinomas [22]. Co-occurring genomic alterations significantly affect the cellular phenotype, clinical outcomes, and therapeutic vulnerabilities of KRAS-mutant cancers. 38% of the human KRAS-mutant lung adenocarcinoma feature an additional loss-of-function mutation in the tumor suppressor *TRP53* [49], which has an overall prevalence of 50–70% in human NSCLC [22].

The first protocol describing the use of a recombinant adenovirus expressing Cre recombinase (adeno-Cre) to induce K-ras G12D expression in mouse lungs was published in 2001 [25], followed by a study in 2005 describing adeno-Cre-mediated activation of K-ras G12D in combination with deletion of p53 [24]. In 2009, Tyler Jacksʼ group published their step-by-step protocol describing their lung cancer KP model [12], which has since been cited over 500 times.

The proof-of-principle experiments in the initial description were based on the intranasal or intratracheal application of both adeno- and lentivirus-based Cre-recombinase to the lung, resulting in the generation of NSCLC [12]. Consequently, the KP model was mainly used to model NSCLC but also to study tumor formation in other organs, e.g., soft tissue sarcomas [14, 29] and pancreatic ductal adenocarcinomas [35]. More recently, normal colon organoids generated from KP mice were transduced with adenoviral Cre, resulting in the generation of colorectal cancer organoids [46]. This indicates that tumor models from different entities could be generated by the in vitro transformation of normal cells derived from KP animals. To allow for autochthonous tumor initiation, the K and P alleles can be combined with an inducible transgenic allele, in which a tissue-specific or inducible promoter regulates Cre-expression, or the combination thereof, as recently reviewed elsewhere [28]. Alternatively, autochthonous tumors can also be initiated spatiotemporally controlled by delivery of the Cre recombinase by viral vectors, e.g., the initial KP lung adenocarcinoma model [12].

### The virus-based delivery of Cre-recombinase typically requires a BSL-2 facility

In the initial study by the Jacks group, both adeno- and lentivirus-based systems were used to deliver Cre recombinase [12]. Due to their potential to enter human cells, deliver genetic material, and elicit an immune response, viral vectors, even when replication-deficient, are typically categorized as a potential biohazard and thus have to be handled at facilities with heightened biosafety measures, i.e., a biosafety level 2 (BSL-2) status. Thus, BSL-2 practices are generally recommended during viral vector administration as it is also recommended by the Viral Vector Core of the University of Iowa (https://vector-core.medicine.uiowa.edu/), e.g., the producer of the adenoviral vector used by the Jacks group in their initial publication [12]. Further, of 44 institutions using viral vectors in animal research, 66% required that animals receiving lentiviral and adenoviral vectors be housed under BLS-2 conditions for several days after administration or the duration of the experiment [10]. Some facilities allow a reclassification to BSL-1 housing after an initial 72 h period of BSL-2 containment [10]. However, in most animal research facilities, animals previously housed in a BSL-2 facility cannot be reclassified to BSL-1 housing because of the significant additional risk that the animals may have been cross-contaminated in a BSL-2 facility. Problematically, a BSL-2 status might restrict access to the equipment required for the noninvasive analysis of tumor growth over time, i.e., micro-computed tomography (micro-CT). In detail, the equipment to perform micro-CT is expensive. Thus, shared animal facilities seek to maximize unrestricted access and house this type of equipment in rooms with BSL-1 status. To address this issue, we sought to replace the virus-based vectors required for Cre recombinase administration with a vector that can be handled in BSL-1 facilities, a gutless, adeno-associated virus (AAV) vector.

### A gutless, adeno-associated virus (AAV) vector for delivery of Cre-recombinase under BSL-1 conditions

Compared to adeno- or lentiviral-based vectors (see also https://www.takarabio.com/learning-centers/gene-function/viral-transduction/recombinant-virus-comparison), vectors based on adeno-associated virus (AAV) feature two main advantages, e.g., decreased immunogenicity and BSL requirements. In detail, adeno- and lentivirus-based vectors trigger a robust humoral and cellular immune response in mice [3, 4]. Anti-AAV humoral responses have also been reported in small animal models. However, although the onset of anti-capsid cellular responses has been observed in several clinical trials, this has never been observed in any of the tested preclinical animal models, even in those susceptible to natural AAV infection, possibly due to difference in the T cell compartment when compared to humans [43].

In the context of biosafety measures, AAV vectors have emerged as one of the most widely used vectors for in vivo gene therapy, e.g., AAV1-based gene therapy for the treatment of lipoprotein lipase deficiency was approved as the first viral vector-based therapy in Europe back in 2012 [41]. The latest generation of recombinant vectors derived from wild-type AAVs and used for gene delivery contains the so-called “gutless” AAV vectors that do not harbor any adenoviral genomic material except for the AAV inverted terminal repeats (ITRS), which serve as a “packaging signal” [45]. According to the NIH guidelines, AAV vectors are classified under risk group 1 (not associated with disease in healthy human adults), given that they do not encode a toxic or potentially harmful transgene, and that appropriate precautions were taken during vector preparation and administration [10].

### Longitudinal monitoring of tumor growth by micro-CT

The original publication of the establishment of the KP tumor model was closely followed by studies showing the feasibility of monitoring this modelʼs tumor formation and growth over time by micro-CT [19] and its tumor response to radiation therapy [30]. Indeed, it was shown that repetitive measurements by micro-CT were not affecting tumor growth over time [17]. Longitudinal monitoring of tumor growth in individual animals of a single animal cohort has several advantages over the analysis of multiple independent cohorts at specific time points. In the first scenario, individual animals can be monitored over time, allowing pairwise statistical analysis of tumor growth. This significantly reduces the number of animals required per experiment and thus has ethical and economic advantages.

In summary, in this report, we present a protocol for adapting the existing KP lung cancer mouse model in which the lentiviral vector is substituted with a gutless AAV vector that allows animals to be handled in a BSL-1 facility. The BSL1 status of the animals allows repeated monitoring of tumor growth over time with a micro-CT device, which is rarely available in BSL2 rooms of animal research facilities. In addition, we optimized the anesthetic protocol and switched to a microscope-guided procedure for vector instillation. This has increased efficiency and significantly reduced complications associated with the process, contributing to compliance with the 3Rs principles.

## Results

### Optimizing the anesthesia protocol and switching to microscope-guided viral instillation increases productivity and reduces procedure-related complications

**Fig. 1:**
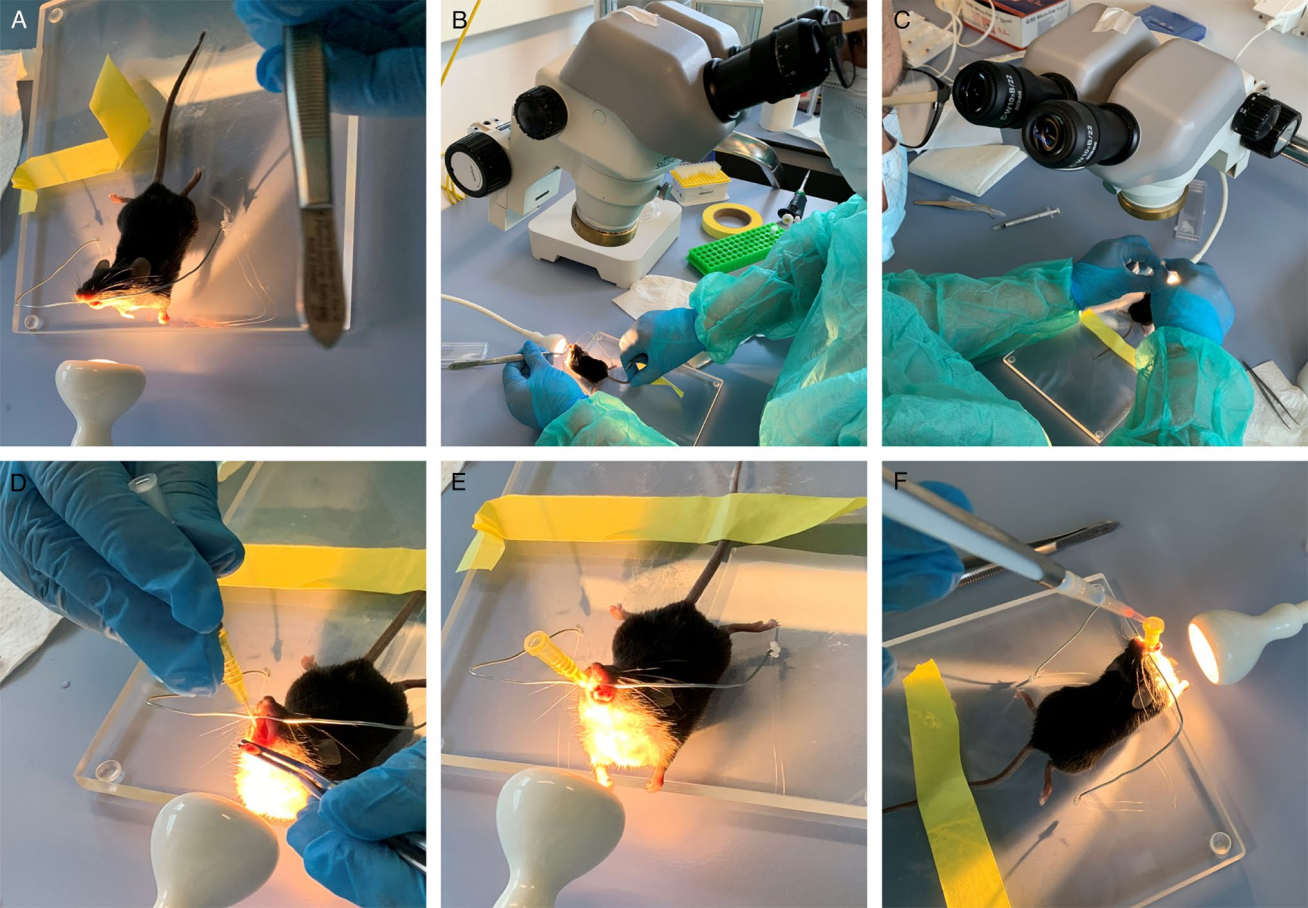
Microscope-guided viral instillation increases productivity and reduces procedure-related complications. Depiction of the procedure for administering infectious solutions by intratracheal instillation based on the original KP protocol by DuPage et al. [12]. As a modification, a stereomicroscope is used to guide the insertion of the cannula and the subsequent steps of inoculation with the infectious solution. The procedure is described in detail under “Administration of viruses/vectors by intratracheal instillation” in the Materials and Methods section

To adapt the original KP protocol [12] to our setting, we implemented two major changes to the protocol. First, we changed the anesthesia protocol to replace tribromoethanol (trade name Avertin®), which is no longer manufactured as a pharmaceutical-grade drug and is also listed as an inadmissible anesthetic in Switzerland (https://www.blv.admin.ch/blv/en/home/suche.html#Tribromoethanol). We first switched to Ketamine/Xylazine-based anesthesia, which was previously regarded as the agent of choice for rodent injectable anesthesia [8]. However, in our hands, this resulted in unstable anesthesia, which made the subsequent intratracheal instillation very difficult. In addition, this protocol resulted in long recovery times as reported before [2], and also animal death. Subsequently, we switched to a Ketalar, Domitor, and Fentanyl-based anesthetic protocol [1]. The Ketalar and Fentanyl-based anesthetic protocol resulted in the rapid onset of deep anesthesia. In addition, the availability of an antidote in the protocol significantly shortened the follow-up time. As a second measure, we began to perform intratracheal instillation using a stereomicroscope (Fig. 1). This allowed us to easily distinguish the trachea illuminated by the flashlight from the esophagus. The adapted anesthetic protocol combined with the microscopy-based inoculation protocol increased our productivity, e.g., routinely instilling 10–12 animals per hour. In addition, we have performed approximately 100 procedures with our adapted protocol to date, but only three animals have died. Thus, in our hands, the two modifications significantly increased productivity and reduced the rate of procedure-related deaths.

### Tumor initiation by a gutless AAV-Cre vector does not alter disease onset or stage in comparison with the AV-based KPL model

**Fig. 2:**
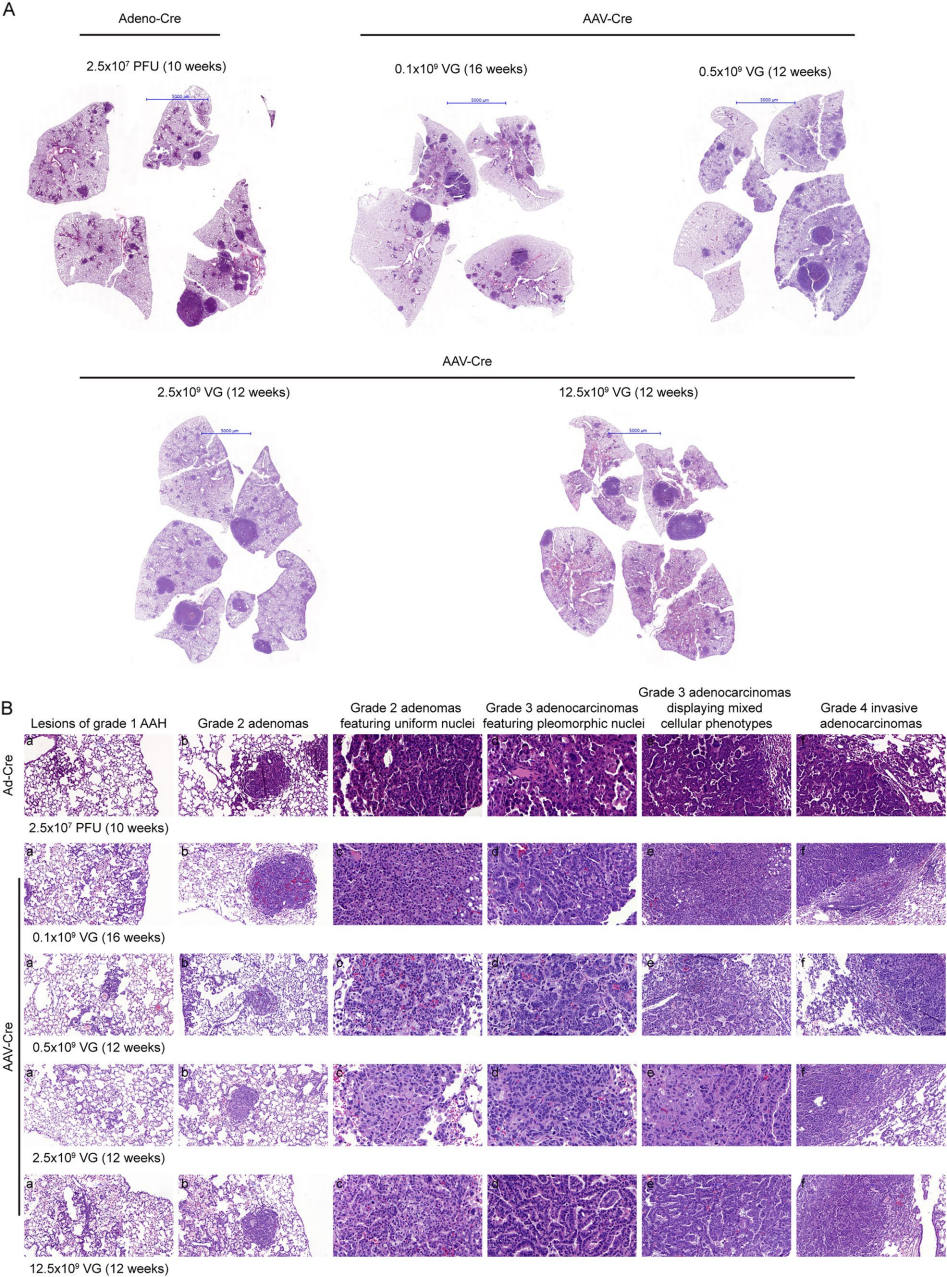
Substituting the adeno-Cre with an AAV-Cre virus results in a similar onset and tumor stage pattern of the disease as observed in the original KP model. **A** Hematoxylin & Eosin (H&E)-stained lung tissue sections after transduction with adeno-Cre and various AAV-Cre titers. Scale bar = 5000 μm. **B** Higher magnification of H&E-stained lung sections featuring different stages of tumor development. Column 1: lesions of grade 1 atypical adenomatous hyperplasia (AAH) progressing to small adenoma, column 2: grade 2 adenomas, column 3: grade 2 adenomas featuring uniform nuclei, column 4: grade 3 adenocarcinomas featuring pleomorphic nuclei, column 5: grade 3 adenocarcinomas displaying mixed cellular phenotypes, and column 6: grade 4 invasive adenocarcinomas

We aimed to optimize the KP model without changing its phenotype. In the initial publication, the Jackʼs group monitored tumor development and progression based on the analysis of histological sections [12]. The KP model results in a multi-focal disease in which individual tumors are not at the same stage of disease development. Thus, the initial study employed a four-stage grading system for tumor progression. 10 weeks after the application of an adeno-Cre virus at a dose of 2.5 × 10^7^ plaque forming units (PFU), we also observed tumors of all four grades, which were detectable after H&E staining of histological sections (Fig. 2A). While a functional titer was determined for the adeno-Cre virus, i.e., the titer was expressed in PFU, a physical titer was determined for the AAV-Cre vector, i.e., viral genomes (VG). It was found that the conversion of PFU to VG was highly variable, e.g., the ratio varied from 1:10 to 1:3600 for the conversion of physical units to biological activity [18]. Therefore, we assumed that the actual biological activity would be 100-fold lower when the titer was determined as VG compared to PFU. Indeed, 12 weeks after the application of the AAV-Cre vector at a dose of 2.5 × 10^9^ viral genomes (VG), a similar tumor burden was observed (analysis after 10 weeks was performed by micro-CT, see Fig. S1). Further, all four stages of tumor progression were detectable in histological H&E sections independently of the virus titer (Fig. 2B).

### The adaptation of the virus titer can control the degree of tumor burden

We titrated the AAV-Cre virus stock to mimic the adeno-Cre-induced tumor development. Indeed, instillation with increasing doses of AAV-Cre virus resulted in increased tumor burden (Fig. 2A). Further, this was also accompanied by an increase in the area with tumor-associated inflammation, e.g., regions of lung tissue with increased H&E staining (Fig. 2A, 0.5 × 10^9^ VG, lung parenchyma of the left compared to the right lower lobe, respectively). In summary, based on the analysis of the histology sections, substituting the adeno-Cre with an AAV-Cre virus resulted in a similar onset and tumor stage pattern of the disease as observed in the original KP model.

### Longitudinal monitoring of KP tumor growth by micro-CT

**Fig. 3:**
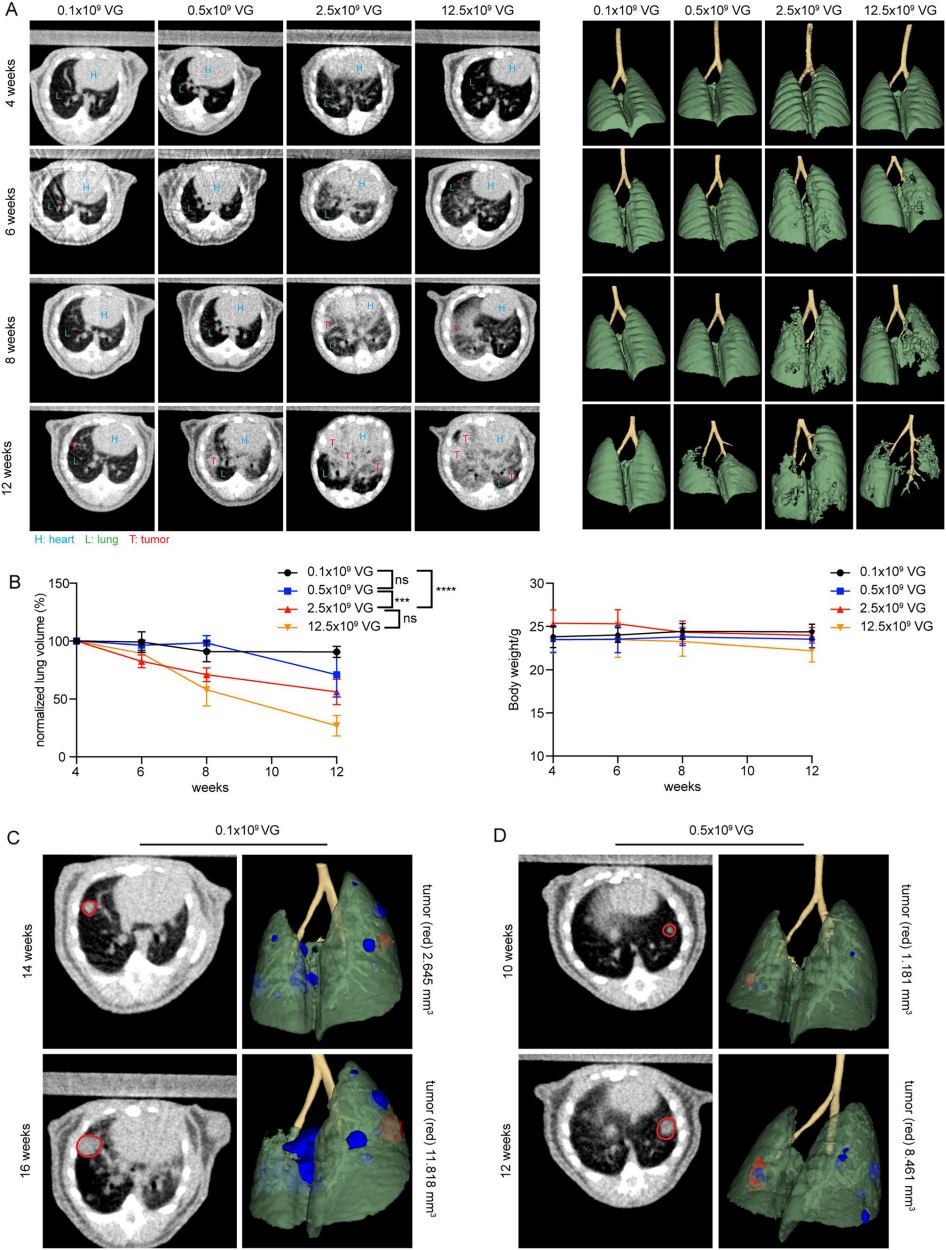
Longitudinal monitoring of tumor growth by micro-CT and 3D reconstruction. **A** Tumor growth was monitored longitudinally by micro-CT after inoculation with the indicated vector titers. The heart (H), lungs (L), and tumors (T) are directly identified on the images by respective labels. (left side). 3D reconstruction of functional lung volume was calculated using the 3D Slicer software (right side). **B** Quantification of normalized lung volume over time of animals after inoculation with the indicated vector titers. Data are presented as mean ± SEM, *n* = 3–4 mice. Two-way ANOVA, multiple comparisons, *ns* no significant difference, ***P < 0.001, ****P < 0.0001. **C** After inoculation with a low vector titer of 0.1 × 10^9^ VG, individual nodules were identifiable by micro-CT and could be followed over time (see red marked nodules). **D** Similar results were obtained after inoculation with a vector titer of 0.5 × 10^9^ VG

The KP model has already been combined with the analysis by micro-CT for longitudinal monitoring of tumor growth per se or after additional radiotherapy [19, 30]. Due to the reduced biosafety requirements of the AAV-Cre vector, we were able to move the animals between the animal holding area and the room where the micro-CT unit was located, both of which were in the BSL-1 zone of our animal facility. This allowed us to monitor tumor growth over time using sequential micro-CT. The analysis by micro-CT revealed that 4 weeks after inoculation with different virus doses, no individual tumor nodules were detectable (Fig. 3). 6 weeks after inoculation with a high virus dose, e.g., 2.5 and 12.5 × 10^9^ VG, respectively, few single nodules became evident. Others showed that 8 weeks after infection of KP animals with a high dose of adeno-Cre virus, regions with a high signal intensity were detectable by micro-CT, and those regions were associated with increased macrophage infiltration [19]. Indeed, large areas with increased signal intensity became noticeable after infection of KP animals with a high dose of adeno-Cre virus (Fig. 3A, 6 weeks, 2.5 and 12.5 × 10^9^ VG).

### Early onset disease with a heavy tumor burden induced by high-titer AAV-Cre vector transduction can be monitored by 3D reconstruction

Due to the multiplicity and complexity of tumor formation induced by a high titer virus infection, identifying and quantifying individual tumor nodules over time is not feasible. Therefore, Haines et al. developed an analytical method to quantitatively measure total lung tumor burden based on micro-CT imaging [19]. In detail, tumor tissue cannot readily be distinguished from vasculature by non-contrast micro-CT since the two tissues have similar X-ray densities [19]. However, the total tumor burden correlates proportionally with the combined tumor and vasculature volume in the lung, which, when subtracted from the total chest space, can be used to calculate the remaining functional lung volume as recently described by us before [11]. Indeed, 8 weeks after inoculation with high virus doses, e.g., 2.5 and 12.5 × 10^9^ VG, respectively, tumor lesions became visually detectable in the 3D reconstruction of the intact lung volume (Fig. 3A, 6 weeks, 2.5 and 12.5 × 10^9^ VG). In the 3D reconstructions, lesions increased visually over time, e.g., at 8 and 12 weeks after inoculation with higher viral doses. After the inoculation with 2.5 and 12.5 × 10^9^ VG, the mean functional lung volume was significantly reduced compared with the group inoculated with a low dose of 0.1 × 10^9^ VG (Fig. 3A, right side, bottom left and bottom right panels, respectively, and Fig. 3B). Interestingly, although significant changes in normal lung volume were detectable over time, this did not yet translate in reduced body weight during the observation period (Fig. 3B).

### Single tumor nodules induced by low-titer AAV-Cre vector transductions can be monitored over time by micro-CT

In humans, lung cancer usually appears as a single tumor nodule at the time of diagnosis. Previously, a single KP tumor nodule was induced by intrathoracic injection of the adeno-Cre virus to recapitulate human disease and serve as a preclinical model for testing treatments for localized disease, e.g., radiotherapy [23]. We observed that after a prolonged latency period following inoculation with a low viral titer, e.g., 0.1 × 10^9^ and 0.5 × 10^9^ VG, respectively, few lesions appeared, and individual nodules became readily detectable by micro-CT (Fig. 3C, D).

**Fig. 4:**
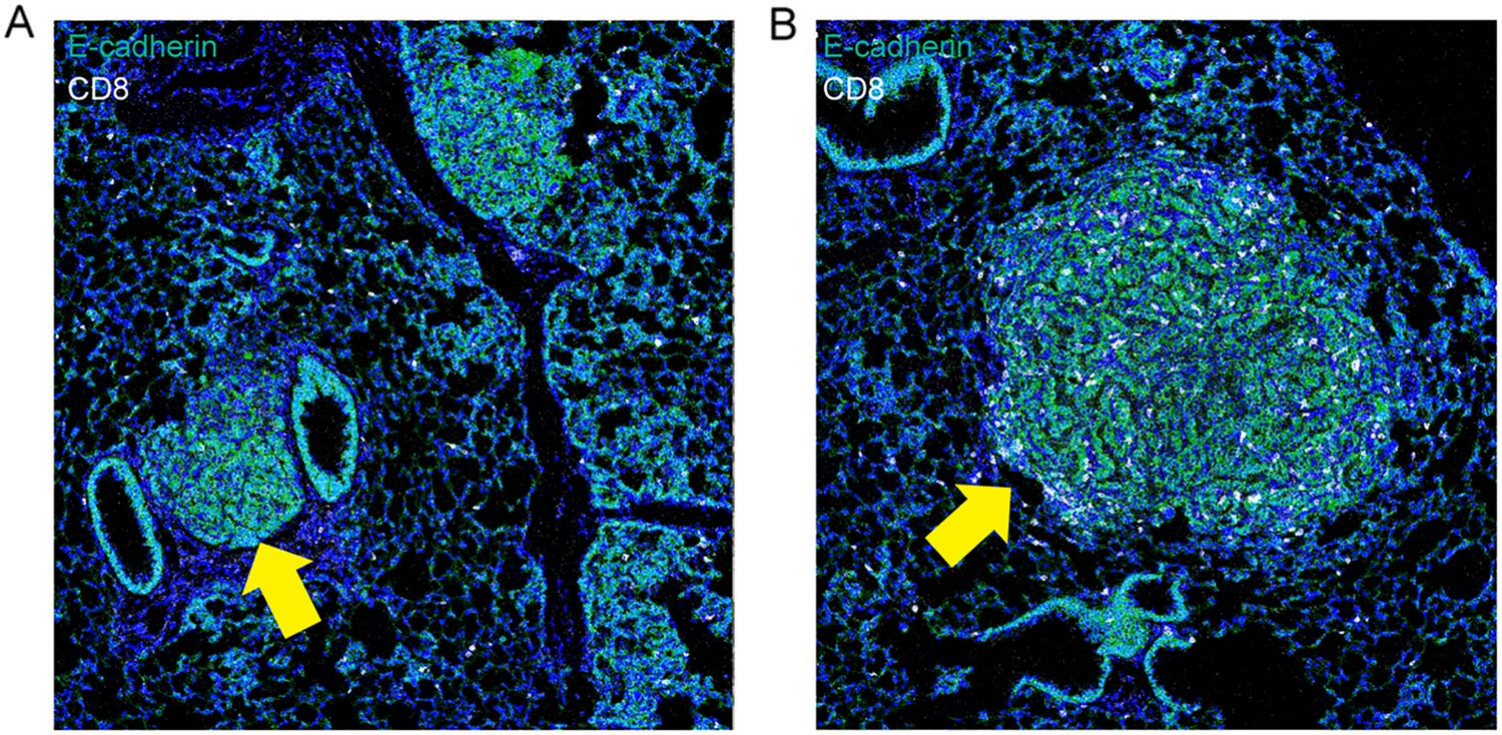
Individually forming tumor nodules are differentially infiltrated by CD8-positive cells after inoculation with a low vector titer. Imaging mass cytometry visualization of two tumor nodules from the same animal 10 weeks after vector instillation. **A** Imaging mass cytometry visualization of E-cadherin (green), CD8 (white), and DNA (blue). **B** Same analysis of a different lesion from the same animal

Finally, analysis of formalin-fixed, paraffin-embedded samples by imaging mass cytometry revealed that individual tumor nodules formed after inoculation with a low viral titer of 0.1 × 10^9^ VG were readily distinguishable by the accumulation of increased E-cadherin signal intensity (Fig. 4). A significant proportion of the nodules did not show increased infiltration with immune cells compared with surrounding lung tissue (Fig. 4A). This is in agreement with previous publications, which indicated that the lack of neoantigens in the KP model results not elicit tumor-specific T cell responses [12, 16]. However, a subset of tumor nodules exhibited significant infiltration by CD8-positive cells, a critical subpopulation of MHC class I-restricted T cells that are mediators of adaptive immunity (Fig. 4B). Infiltration with CD8-positive cells was often accompanied by infiltration of the immediate nodal environment by CD206-positive cells (Fig. S2), e.g., a marker of activated tumor-associated macrophages associated with enhancement of the adaptive and innate antitumor immune responses [26]. Because the AAV vector used in our study does not lead to the expression of neoantigens associated with lentiviral vector integration [13], it will be interesting to investigate what drives the observed inflammation in a subset of tumor nodules induced by AAV-Cre transduction in the KP lung cancer model.

## Discussion

In this study, we implemented several changes to the original KP tumor model. Regarding the biosafety aspects of our protocol adaptations: the latest generation of “gutless” AAV vectors contains no adenoviral genomic material except for the adenoviral packaging signal. They are thus entirely replication-deficient and cannot be restored by recombination events, which pose a biohazard thread during the production of lentiviral particles. Thus, AAV particles, in the absence of a toxic or hazardous transgene, can be handled at BSL-1 facilities. Cre-recombinase is not considered a biohazard, according to NIH guidelines. In addition, the Cre-expressing AAV used in our study is commercially available (EPFLʼs PTBTG gene therapy platform (https://www.epfl.ch/research/facilities/gene-therapy/), which facilitates the transition to the adapted protocol for other research groups.

In our current version of the protocol, CaCl_2_ is added to a final concentration of 10 mM because it has been shown that incorporation of AAV into a calcium-phosphate coprecipitate enhances gene transfer to airway epithelia in vitro and in vivo [44]. However, a recent publication indicated that the addition of CaCl_2_ could actually inhibit the transduction efficiency of AAV2 vectors, whereas the combined use of cobalt and zinc resulted in a more than 30-fold increased transduction efficiency of AAV2 vectors [37]. From a pragmatic perspective, although modifications to the transduction protocol could reduce costs by decreasing the amount of viral solution required, the adapted protocol would need to be re-titrated to achieve the desired number of tumors.

Previously, it has been shown that total tumor and vascular volume increase over time in a dose-dependent manner in the KP lung cancer model [19]. Specifically, it was reported that during a 12-week period, beginning at week 8, total tumor and vascular volume increased approximately twofold and fivefold, respectively, following induction with a low and a high AV-Cre vector titer. Consistent with this, normalized lung volume was also reduced approximately fourfold over an eight-week period beginning at week 4 following induction with a high AAV-Cre vector titer. Thus, the AV- and AAV-based vectors resulted in comparable disease progression, but further experiments would be required to determine the relative proportion of each stage at different time points in the two models. Based on the analysis of lung H&E sections, quantifying the total number of tumor nodules per animal was challenging since the tumor stage of the individual nodules was very heterogeneous. In detail, determining the individual tumor stage of the single nodules requires the expertise of a trained pathologist. Thus, our study reported a qualitative analysis of the tumor stage, which showed that substituting the lentiviral vector with the AAV-Cre vector did not result in a dramatic change in the tumor stage distribution. The exact tumor number per vector dose was not reported in the original lung KP protocol, which concluded that tumor numbers and time to progression of autochthonous mouse tumor models vary widely depending on the strain/background of the mice as well as other factors that vary from institution to institution [12]. Thus, it would be interesting in future studies to compare the detailed disease status and progression induced by the AV- and AAV-based systems and to determine the exact tumor-inducing capacity of the novel AAV-Cre vector in extreme limiting dilution assays.

In a study aimed at comparing the effect of different treatment regimens on tumor growth, the number of animals required for statistically significant results can be reduced by minimizing differences in tumor burden between animals at the start of treatment. We observed significant differences in tumor burden between animals 8 weeks after the instillation with a high virus titer (Fig. S1). Thus, micro-CT-guided individualization of the time point of treatment initiation would allow for a reduction in differences not only in disease burden but also in the associated tumor stage, which changes during disease progression.

In tumor models, the use of humane endpoints allows for early termination of experiments and minimizes animal discomfort, suffering, and pain while ensuring that scientific goals are met [36]. Based on previously published protocols [11, 19, 48], our study provides an open-source protocol for analyzing micro-CT data to monitor tumor growth over time by quantifying “net change in lung volume.” The drop in lung volume over time preceded the loss of body weight (Fig. 3B), which is also considered a humane endpoint [36]. Ideally, to determine whether our micro-CT protocol is appropriate for measuring tumor growth over time, a benchmark comparison with other methods should be performed, such as determining tumor growth by immunohistochemistry at various endpoints. Nevertheless, the quantification of “net change in lung volume” might be well suited for the early identification of animals harboring a high tumor load and thus are at a higher risk of suffering or death thereby clearly contributing to compliance with the 3Rs principles.

A shortcoming of our study is that we did not benchmark our CT-based protocol to determine tumor growth over time. Further, we did not explicitly test whether Cre is still expressed in the induced tumors. In detail, it has been shown that the constitutive endonuclease activity of Cre significantly reduces the proliferation of mammalian cells [32] and that the inducible expression of Cre induced regression of primary lymphomas in p53-deficient mice [31]. However, gutless AAV vectors are unable to integrate and their cargo DNA is lost over time as their transgenes exist in the host cell nucleus as non-replicating episomes [9, 20]. However, in the context of using the KP model to study immunotherapy [16], it would be of interest to observe in subsequent studies how Cre is expressed in the KP model over time. In addition, a variety of serotypes are available for AAV-based vectors, which could serve as unique tools to further study tumorigenesis and lung cancer development in the KP model [34] in the absence of the immune response induced by the adeno-Cre-based system [38]. In the context of the immune response, further studies will be necessary to examine whether the AAV-based vectors also infect immune cells, particularly macrophages resident in the lung microenvironment as it was shown previously for adeno-Cre-based viruses [40]. Finally, it would be interesting to characterize in detail how the serotype and capsid choice affect the cellular tropism of AAV-based vectors and consequently the tumor-associated immune response.

## Conclusions

In summary, by substituting the adenoviral/lenti-Cre vector with an AAV-Cre vector, our adapted protocol allows handling the KP mouse model under BSL-1 conditions so that virus-transduced animals can be repeatedly analyzed by micro-CT, thereby drastically reducing the number of animals required for longitudinal monitoring of tumor growth. Also, in compliance with the 3R principle, optimizing the anesthesia protocol and switching to microscope-guided viral instillation increases productivity and reduces procedure-related complications. Finally, our user-friendly protocol for 3D reconstruction of tumor volume using open-source software will further foster the broad applicability of the KP tumor model.

## Methods

Intratracheal delivery of a viral vector encoding Cre recombinase into the Kras-Lox-STOP-Lox-G12D p53 flox/flox (KP) mouse model faithfully recapitulates human lung adenocarcinoma. Handling virus-based delivery systems requires elevated biosafety measures, e.g., a biosafety level 2 (BSL-2) infrastructure. However, in animal research facilities, small animal micro-computed tomography (micro-CT) equipment is typically stored in general access areas, e.g., in BSL-1 compartments. Our goal was to adapt the protocol so that the Cre-induced KP mouse model could be handled during the entire procedure under BSL-1 conditions, allowing repeated micro-CT analyses of individual animals, thereby dramatically reducing the number of animals required for longitudinal monitoring of tumor growth.

### Mouse model

The Kras^LSL-G12D/+^;Trp53^fl/fl^ mice were bred and obtained from our in-house animal facility at the University of Bern, Switzerland. In detail, Kras^LSL-G12D/+^;Trp53^fl/fl^ mice were generated by crossing stock B6.129SS4-krastm4Tyj/J mice with B6.129P2-Trp53tm1Brn/J [25, 33]. All animal experiments were carried out in accordance with protocols approved by the local ethics committee of the Canton of Bern; license number PB_2016-01560 and BE49_2022.

### Anesthesia

The mouse experiments were performed in accordance with the animal welfare guidelines and protocols approved by the institutional animal care and ethical committee, i.e., the ethic commission of the Canton of Bern, Switzerland, license number BE49_2022. The protocol was developed based on a publication by Henke and Erhardt [15], whereas the composition of the fully reversible anesthesia was based on a publication by Henke and Müller [21]. In detail, mice at 8 weeks of age were anesthetized with a solution containing 30% Ketalar (Pfizer, Cat. No. 35073), 20% Domitor (Orion, Cat. No. 50590), and 12.7% fentanyl (Mepha, cat. no. 53987) in 0.9% Sodium chloride (NaCl, B. Braun, Cat. No. 3570130) by intraperitoneal (i.p.) injection [1]. After vector instillation, mice were immediately administered an antagonist solution containing 10% Revertor (Virbac, Cat. No. 58701) and 60% Naloxone (Mepha, Cat. No. 56952) in 0.9% NaCl by i.p. injection. This anesthetic protocol avoids unnecessary stress to the animal and allows very regular breathing throughout the procedure, allowing smooth intubation of the animals without accidental ingestion of the virus suspension. Further details are given in the ‘Step-by-step procedure’ section.

### Vector information and preparation of virus-containing solutions

AAV2/9-cmv-Cre: the genome is flanked by the inverted terminal repeats of AAV serotype 2 and is packaged in the capsid of AAV serotype 9. Transcription of recombinant Cre recombinase is driven by a cytomegalovirus (CMV) promoter. AAV2/9-cmv-Cre was produced by the Bertarelli Foundation Gene Therapy Platform at the Swiss Federal Institute of Technology, Lausanne (https://www.epfl.ch/research/facilities/gene-therapy/. The platform can customize any construct with one or more sequences of interest and/or perform complete design and cloning of the transfer construct. A wide range of vector backbones (AAVs and lentiviruses) are available, including a variety of promoters, reporter genes, and enhancers). Specifically, the high titer and high purity AAV vector batches were generated by transient transfection with a two-plasmid system of mammalian cells (HEK293) grown in orbitally shaken suspension cultures as previously described [7]. The original vector was freshly diluted with Minimal Essential Media (MEM, Sigma-Aldrich, cat. no. M8042) to various titers. Then, CaCl_2_ was added to a final concentration of 10 mM, and the solutions were incubated for 20 min at room temperature.

Ad5CMVCre: adenoviral vector with CMV (Cytomegalovirus) promoter driving the expression of the Cre Recombinase protein (https://vector-core.medicine.uiowa.edu/products/ad5cmvcre?variant=40576502096). Ad5CMVCre was purchased from the University of Iowa, Gene Transfer Vector Core.

Preparation and administration of virus-containing solutions should occur in a biosafety hood and follow all guidelines for BSL-2 research.

### Vector administration

Solutions containing vectors at various concentrations were administered to animals by intratracheal instillation monitored through a stereomicroscope (Nikon SMZ645, zoom range of 0.8×–5× magnification). The technical details are described in the “Step-by-step procedure” section. See also the supplementary video of the publication of DuPage et al. [12].

### micro-CT scan and image analysis

Beginning 2 weeks after vector instillation, lungs were examined weekly using an X-RAD SmART Precision X-Ray Imaging System (Precision X-Ray, North Branford, CT) to confirm tumor formation. In detail, mice were continuously anesthetized with 3% isoflurane. Photons were filtered using a 2 mm Al filter for computed tomography. The raw DICOM data were imported into the 3D slicer for visualization and quantitative analysis [42]. For lung volume analysis, automatic segmentation of the lungs and airways was performed based on the “Grow from Seeds” tool. Subsequently, the “Segment Statistics” tool was used to quantify lung volume. For tumor volume analysis, the window and level in “Volumes” were adjusted to better separate lung and tumor. Subsequently, tumors were segmented in “Segmentation Editor” and quantified using “Segment statistics.” After segmentation, 3D reconstruction was performed.

### Immunohistochemistry

Hematoxylin & Eosin (H&E) staining was performed as previously described [5].

### Imaging mass cytometry

Imaging mass cytometry (IMC) was performed using an optimized mouse antibody panel for FFPE tissues [6] In brief, 3 μm sections of FFPE mouse lung tissues were sectioned and dried overnight. Antigen retrieval, blocking and staining with metal-labeled antibodies were performed the following day (use same reference as above). On day three, DNA was stained with intercalator-Ir (iridium) and the sections were acquired on the Hyperion tissue imager (Standard BioTools) at the University of Bern/Inselspital IMC platform (www.imc.unibe.ch). The mass cytometry data (MCD) file was loaded into napari: multi-dimensional image viewer for python (Sofroniew [39]) and the napari-imc plugin was downloaded and employed [47]. We visualized the channels for DNA in blue, E-cadherin (tumor) in green, and CD8 (cytotoxic T cells) in white (Fig. 4).

### Statistical analysis

Statistical analysis was performed using GraphPad Prism 9. Results were collected from at least three different mice. Error bars represent the mean ± standard error of the mean (SEM). Two-way ANOVA was performed as described in the figure legends. The *p* values < 0.05 were considered significant. In all analyses, the significance level is indicated as follows: *P < 0.05, **P < 0.01, ***P < 0.001, ****P < 0.0001.

### Step-by-step procedures

#### Necessary equipment

**Table:** 

1 mL syringes and needles	Hanging tray
Pipette P100	Rubber air blower
Pipette P10	Forceps
Pipette tips	Lamp
Cannula 20 G and 18 G	Microscope (0.8×–5× magnification)
Heating pad	

Prepare anesthesia as a batch solution (*n* + 1), and keep it at room temperature. Prepare 100 μl per animal:

#### Anesthesia solution

- 300 μl Ketalar 50 mg/ml
- 200 μl Domitor 1 mg/ml
- 127 μl Fentanyl 0.05 mg/ml
- 373 μl NaCl·0.9%

#### Antagonist solution

- 100 μl Revertor 5 mg/ml
- 600 μl Naloxone 0.4 mg/ml
- 300 μl NaCl 0.9%

#### Virus solution

- Virus solution x μl virus + y μl MEM + 2.5 μl CaCl_2_·0.2 M
- Total volume 50 μl

Prepare the virus solution in a total volume of 50 μl per animal, and adjust the concentration accordingly (Record details in the lab book).

#### Anesthesia (timing: 10 min per cage of 5 mice)

1. Anesthetize the animal by intraperitoneal (i.p.) injection of 80 μl of the prepared anesthetic solution (regardless of differences in body weight).
2. Confirm that the mice are fully anesthetized by ensuring they lack a toe reflex.
3. If not, inject another 20 μl of the anesthetic solution, i.p.
4. Place the animal on its stomach on the heating pad.
5. Apply Bepanthen eye ointment to both eyes.

#### Virus/Vector delivery by intratracheal instillation (Timing: 2–5 min per mouse)

6. Carefully position the animal by the upper teeth in the hanging rack (Fig. 1A).
7. Place the lamp directly on the neck of the animal (Fig. 1A).
8. Fix the tail of the animal with a small piece of adhesive tape.
9. Open the mouth gently with a small forceps using the right hand.
10. Use a second forceps equipped with rubber end caps to gently pull out the tongue with the left hand to the left side (Fig. 1B).
11. Use the stereomicroscope to identify the trachea (pulsating in the center) (Fig. 1C).
12. To fully open the mouth, adjust the tailʼs position by moving the adhesive tape (Fig. 1C).
13. Fully open the trachea by slightly adjusting the forceps.
14. Insert the cannula (attached to the catheter) into the trachea (Fig. 1D). There should be no resistance while inserting the catheter into the trachea.
15. Remove the cannula from the catheter.
16. Place a rubber air blower on the catheter, administer an air bolus, and check if the lungs expand. If the lungs are not expanding, the catheter might be in the esophagus. In this case, gently remove the catheter and repeat the two previous steps. Repeat until the lungs expand visually after applying a bolus of air (Fig. 1E).
17. Pipette the virus solution directly into the opening of the catheter to ensure the entire volume is inhaled (Fig. 1F).
18. When the catheter is correctly inserted into the trachea, the mouse immediately begins to inhale the solution. Once the solution is no longer visible in the opening of the catheter, wait a few seconds for all of the solution to run down the catheter.
19. Add an air bolus with a rubber air blower.
20. Remove the catheter from the trachea.
21. Immediately remove the animal from the hanging rack.
22. Place the mouse on its side on the heat pad.
23. Open its mouth with forceps to facilitate breathing.

#### Animal recovery (Timing: 10 min recovery time)

24. Inject 0.1 ml of the antagonist solution i.p. (do not delay this step).
25. Leave the mouse on the warming pad until fully awakened.
26. Put the mouse back in the cage.
27. Monitor the animal until breathing becomes steady again.
28. Add the “omnivore” sign if necessary.
29. Place the cage back in the animal room.
30. Check the animal the next day (or in the afternoon if anesthesia was performed in the morning).

### Supplementary Information

Below is the link to the electronic supplementary material.

**Figure S1**. Longitudinal monitoring of tumor growth by micro-CT and 3D reconstruction. A. Additional time points for Fig. 3A. B. Micro-CT images of additional animals after inoculation with low vector titers for Fig. 3A.

**Figure S1**. C. Same as in B, after inoculation with high vector titers

**Figure S2**. Imaging mass cytometry visualization of the indicated additional markers of the tumor nodule shown in Fig. 4

## Data Availability

All data generated or analyzed during this study are included in this published article and its supplementary information files.

## Abbreviations

BSL-2: Biosafety level 2
H&E: Hematoxylin & Eosin
KP: Kras-Lox-STOP-Lox-G12D/p53 flox/flox
micro-CT: Micro-computed tomography
NSCLC: Non-small cell lung cancer
PFU: Plaque forming units
