# Supplementary figures and images for: An optimized protocol for the generation and monitoring of conditional orthotopic lung cancer in the KP mouse model using an adeno-associated virus vector compatible with biosafety level 1
Source: Cancer Immunol Immunother. 2023 Oct 5;72(12):4457–70. doi: 10.1007/s00262-023-03542-z (PMC10700219; doi:10.1007/s00262-023-03542-z)

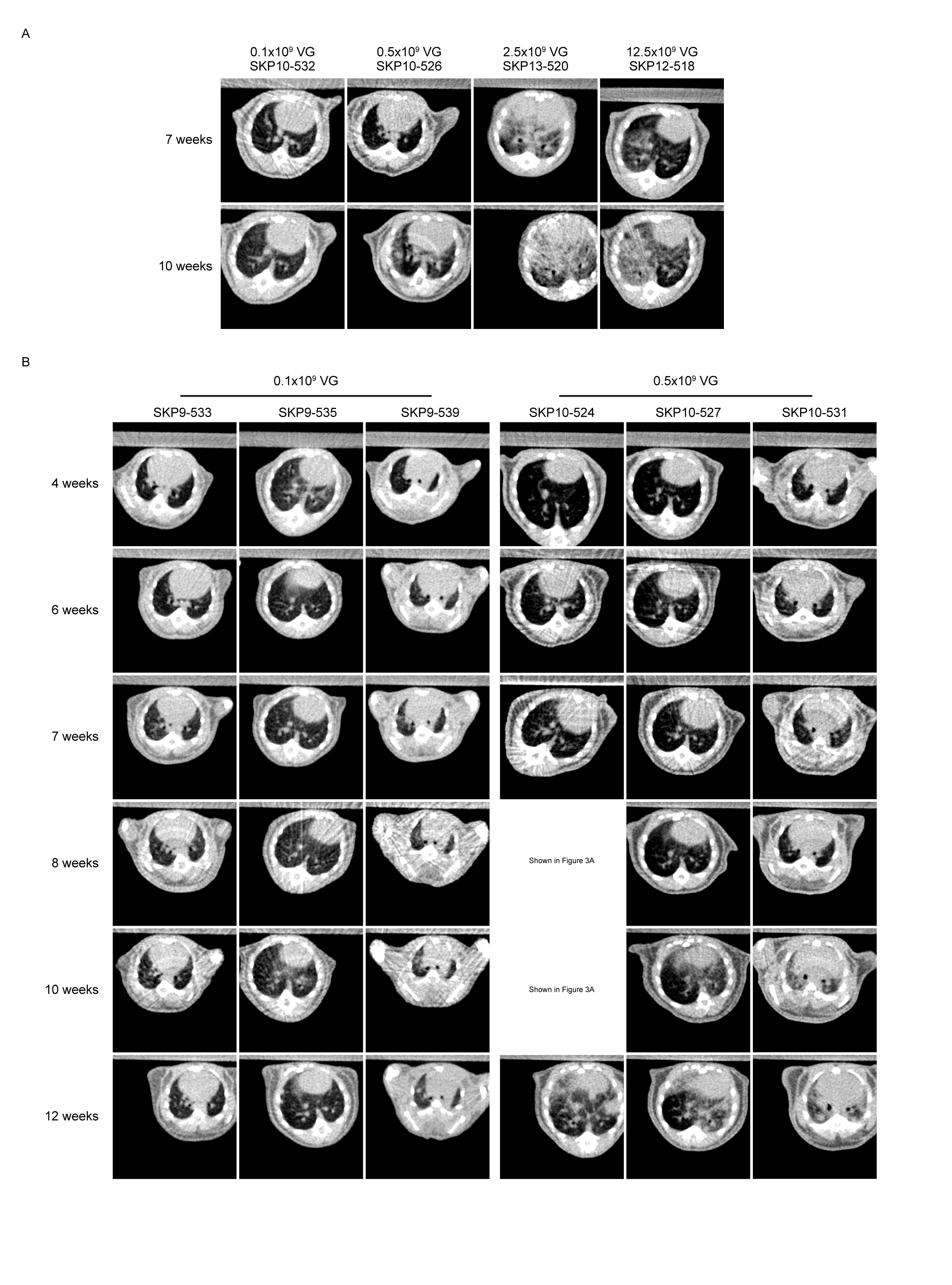

Supplement: Supplementary file 1 — Figure S1. Longitudinal monitoring of tumor growth by micro-CT and 3D reconstruction. A. Additional time points for Fig. 3A. B. Micro-CT images of additional animals after inoculation with low vector titers for Fig. 3A. [file 262_2023_3542_MOESM1_ESM.tif]

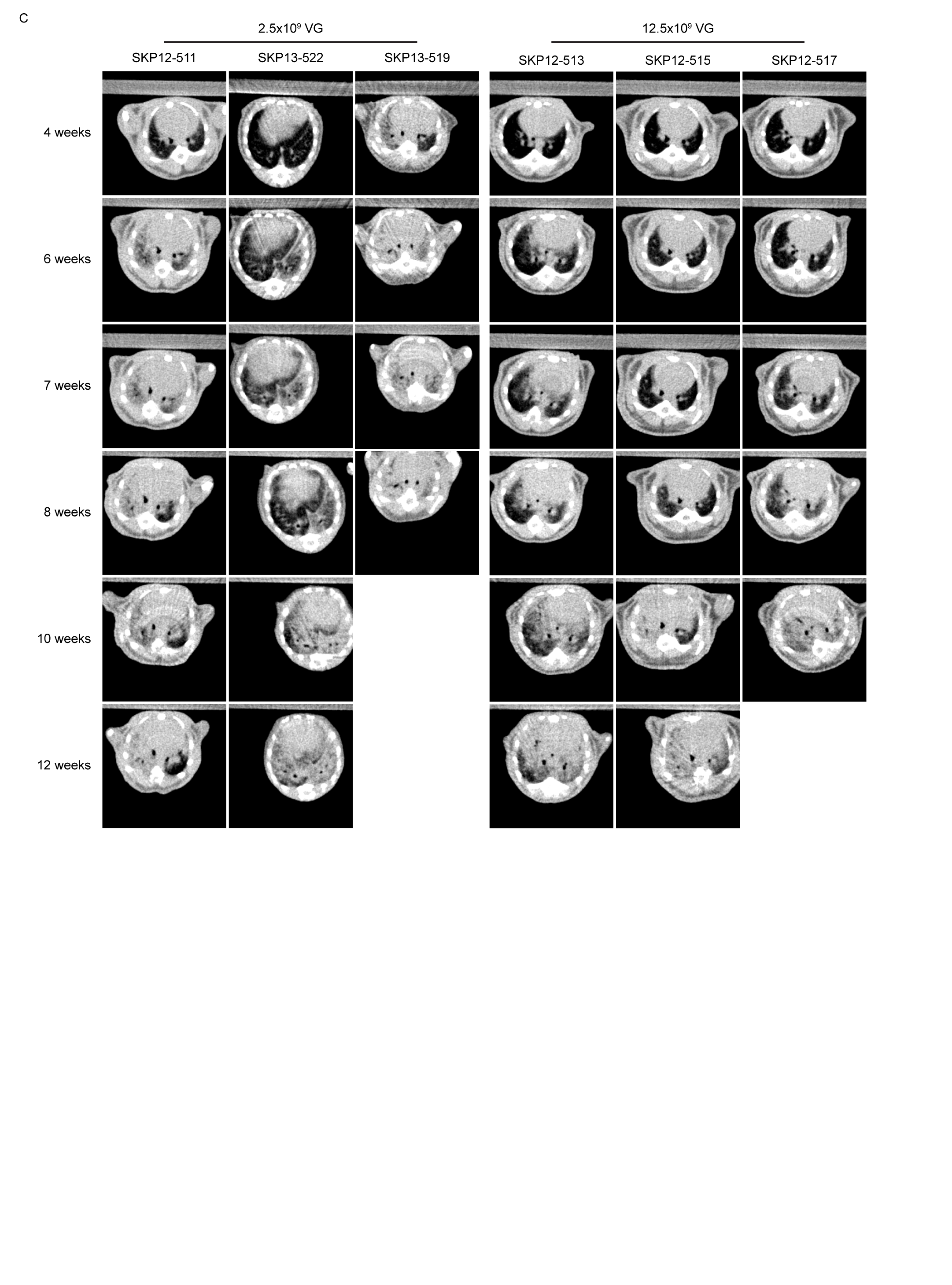

Supplement: Supplementary file 2 — Figure S1. C. Same as in B, after inoculation with high vector titers [file 262_2023_3542_MOESM2_ESM.tif]

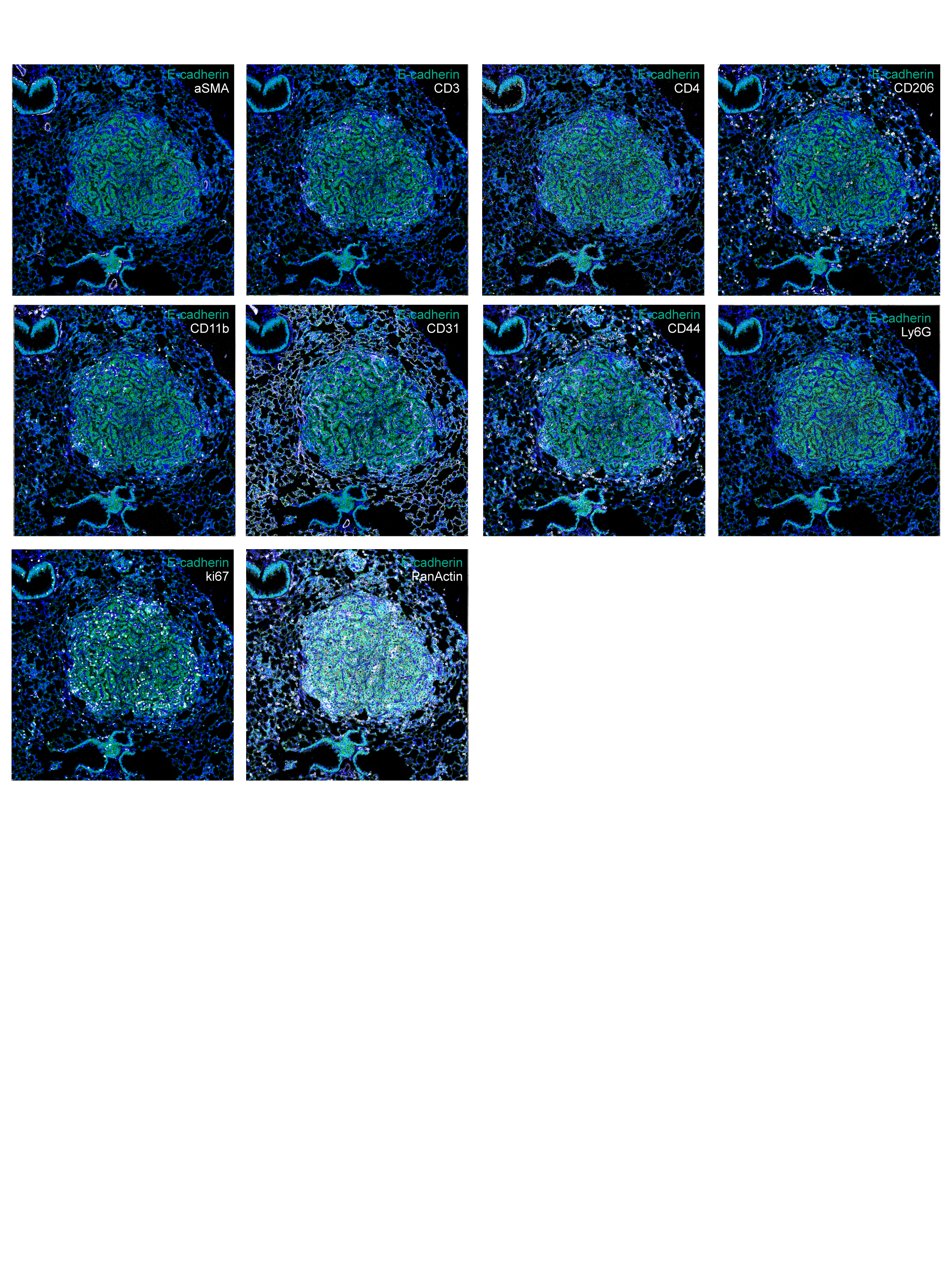

Supplement: Supplementary file 3 — Figure S2. Imaging mass cytometry visualization of the indicated additional markers of the tumor nodule shown in Fig. 4 [file 262_2023_3542_MOESM3_ESM.tif]
